# Degradation of Resistant α-1,4-glucan by Vaginal *Gardnerella* Species is Associated with Bacterial Vaginosis

**DOI:** 10.1007/s00284-025-04459-9

**Published:** 2025-09-02

**Authors:** Rosanne Hertzberger, Sara Morselli, Sara Botschuijver, Lisa Himschoot, Leon Steenbergen, Sylvia Bruisten, Warren Lewis, Piet Cools, Remco Kort

**Affiliations:** 1 Amsterdam Institute for Life and Environment (A-LIFE), Vrije Universiteit Amsterdam, Amsterdam, The Netherlands; 2 Washington University School of Medicine, St Louis, MO USA; 3 Department of Medical and Surgical Sciences, University of Bologna, Bologna, Italy; 4 Department of Diagnostic Sciences, Faculty of Medicine and Health Sciences, Ghent University, Ghent, Belgium; 5 Public Health Laboratory, Department of Infectious Diseases, GGD, Amsterdam, The Netherlands; 6 ARTIS-Micropia, Amsterdam, The Netherlands; 7 Department of Obstetrics, Gynecology, and Reproductive Sciences, University of California San Diego, San Diego, CA USA

## Abstract

This study investigates the degradation of resistant α-1,4-glucan by vaginal bacterial species, with a focus on *Gardnerella* spp., to elucidate its role in bacterial vaginosis (BV). The ability of *Gardnerella vaginalis*, *Gardnerella swidsinskii*, *Gardnerella leopoldii*, *Gardnerella piotii*, *Lactobacillus iners*, and *Lactobacillus crispatus* was assessed to metabolize an ungelatinized, labeled form of raw amylose, a degradation-resistant α-1,4-glucan. The enzymatic activity of these species was evaluated in vitro, and its association with BV was examined in vaginal swabs. *Gardnerella vaginalis, G. swidsinskii*, and *G. leopoldii* demonstrated the best ability to degrade resistant α-1,4-glucan in vitro. Unlike the cell-bound, S-layer-associated glycogen-degrading activity in *L. crispatus*, this α-glucosidase activity in *Gardnerella* was also extracellular, but not cell-bound and not repressed by glucose. Vaginal swabs showing high rates of resistant α-1,4-glucan degradation activity were associated with BV, particularly in the concurrent presence of *G. leopoldii, G. swidsinskii*, and *G. vaginalis*. These findings suggest a role of α-1,4-glucan degradation in BV pathogenesis mediated by *Gardnerella* species. The results indicate the potential of targeting bacterial amylase activity as therapeutic strategy for BV prevention and treatment.

**Supplementary Information:**

The online version contains supplementary material available at 10.1007/s00284-025-04459-9.

## Introduction

The vaginal mucosa of reproductive aged women is rich in glycogen [1, 2]. Through shedding and lysis of the superficial vaginal epithelial cells, glycogen is released in the vaginal lumen where it can serve as a carbohydrate source for bacteria colonizing the vagina, including *Lactobacillus crispatus* [3, 4]. Glycogen levels were found to be associated with the vaginal microbiota composition [5]. A *Lactobacillus*-dominated vaginal microbiota is associated with higher levels of glycogen, whereas a more diverse *Lactobacillus*-depleted microbiota is associated with higher levels of fastidious anaerobes such as *Gardnerella*, *Fannyhessea*, and *Prevotella* [6]. The latter microbial dysbiotic state, often associated with bacterial vaginosis (BV), is a recognized risk factor for adverse reproductive and sexual health outcomes, including HIV infection [7], HPV infection [8], subfertility [9], endometritis [10], pregnancy loss [11], and preterm birth [12].

The vaginal microbiota in women with BV has a much higher bacterial diversity compared to the healthy, *Lactobacillus-*dominated microbiota. Species within the *Gardnerella* genus can cause many of the clinical manifestations of BV, including exfoliation of the epithelial wall lining the vagina and thinning of the mucus leading to abnormal secretions [13–15]. *Gardnerella* spp. differ in their ability to produce sialidase found in *G. piotii, G. pickettii* and in a subset of *G. vaginalis* strains [16, 17]*.*

Alpha-glucosidases are enzymes that catalyze the hydrolysis of α−1,4 or α−1,6 linkages between glucose units of α-glucans. Such amylolytic enzymes belong to the α-amylase family of glycoside hydrolases, primarily classified in CAZy families GH13 and GH57, and include α-amylases, pullulanases, and other starch-degrading enzymes with distinct specificities [18–20]. Moreover, many of these enzymes possess specialized carbohydrate-binding modules (CBMs) that facilitate binding to insoluble glycogen or starch granules, enhancing the breakdown of these resistant substrates [21]. In an environment with glycogen as the prime carbohydrate source, α-glucosidases of vaginal bacteria could play a central role in carbon and energy metabolism by extracellular degradation of host-associated glycogen, thereby releasing smaller maltodextrins for utilization in the surrounding environment. Previously, we have suggested a genetic locus for one of these α-glucosidases: the *L. crispatus* amylopullulanase [3, 4, 6]. Strikingly, we found a large number of *L. crispatus* laboratory strains (23%) to be deficient for this amylopullulanase and identified frequently occurring mutations in the genes of these strains as well as in databases of vaginal metagenomes [3, 22]. This suggests that carbohydrate availability for some strains may depend on glycogen cleavage by other bacteria or host amylase.

Most studies characterizing the α-glucosidases of vaginal bacteria have used soluble starch or glycogen [23–25]. However, for the gut microbiome it was shown that more resistant structures of α-glucan allow to discriminate between carbohydrate-degrading enzymes of different bacterial taxa: Animals fed with degradation-resistant α-glucan (resulting from a denser structure and a lower degree of branching) show an increase of amylolytic species such as *Bifidobacterium* spp. [26, 27]. These bacteria produce a type II amylase-pullulanase fusion protein capable of hydrolyzing resistant starch [28, 29]. A homologue of this enzyme was identified in *Gardnerella vaginalis*, expressed in *Escherichia coli*, and found to degrade amylose, pullulan, glycogen, and starch [30]. More recent research showed that 14 out of 15 tested *Gardnerella* strains harbor a full copy of this gene [23] and that isolates of four different *Gardnerella* species can transport and metabolize the small maltodextrins derived from glycogen breakdown [31].

Here, we aimed to use resistant α−1,4-glucan to distinguish between bacterial glycosidases, thereby increasing our understanding of their contribution to glycogen breakdown in various microbial contexts. We show that *Gardnerella* species can be distinguished from vaginal lactobacilli by their ability to break down resistant α−1,4-glucan. The degradation of resistant α−1,4-glucan appears to be associated with vaginal dysbiosis.

## Methods

### Bacterial Cultivation

**Fig. 1:**
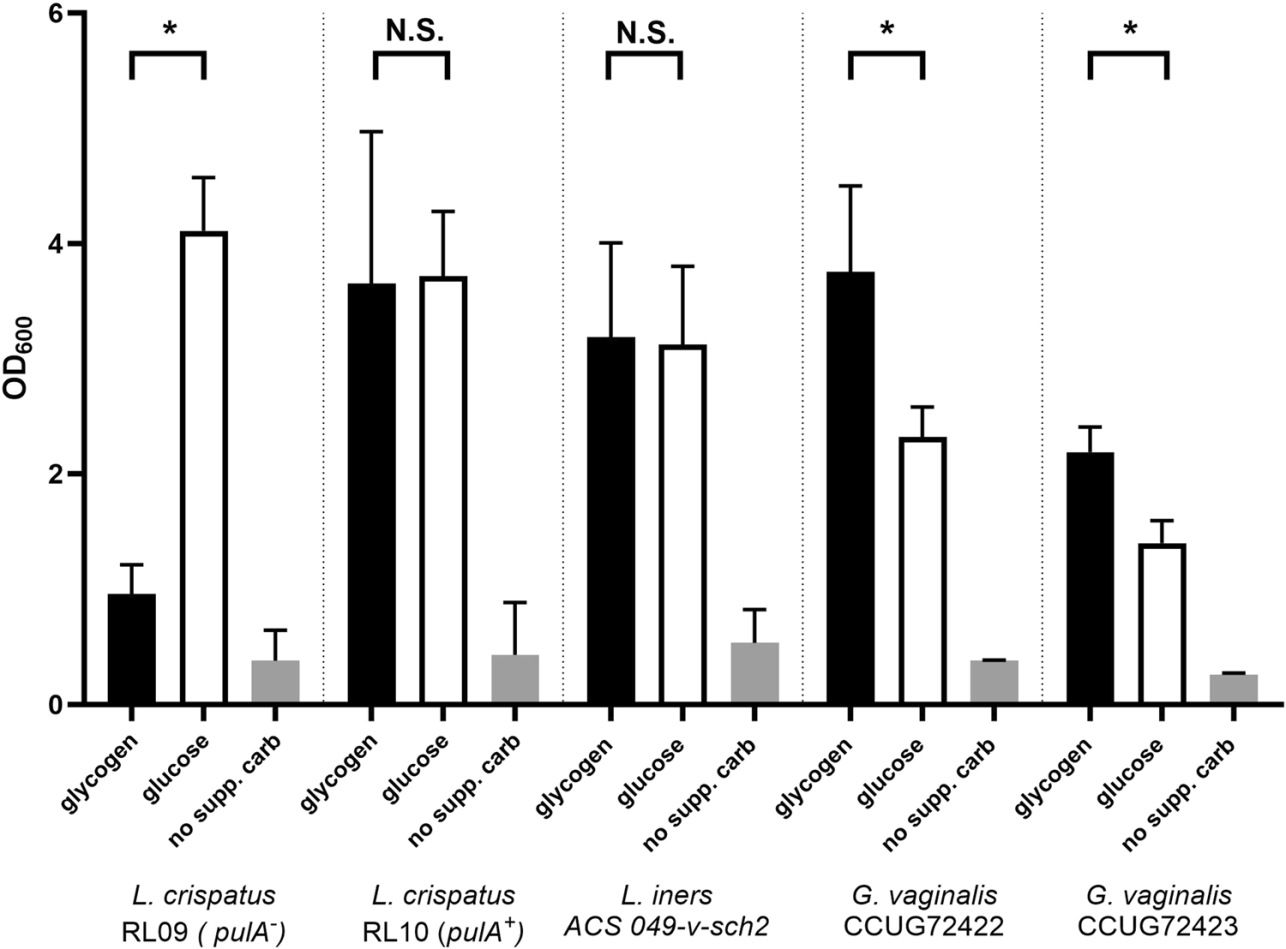
Optical density at 600 nm after 48 h of growth in liquid NYCIII medium with 5 g/L glucose (white bars), 5 g/L glycogen (black bars) or no supplemented carbohydrate source (gray bars). All measurements were performed in three biological replicates in separate experiments on separate days. Data represent average and error bars are the standard deviation. Paired t test was used to compare averages between glycogen and glucose. * = *P* < 0.05; *N.S.* not significant

**Fig. 2:**
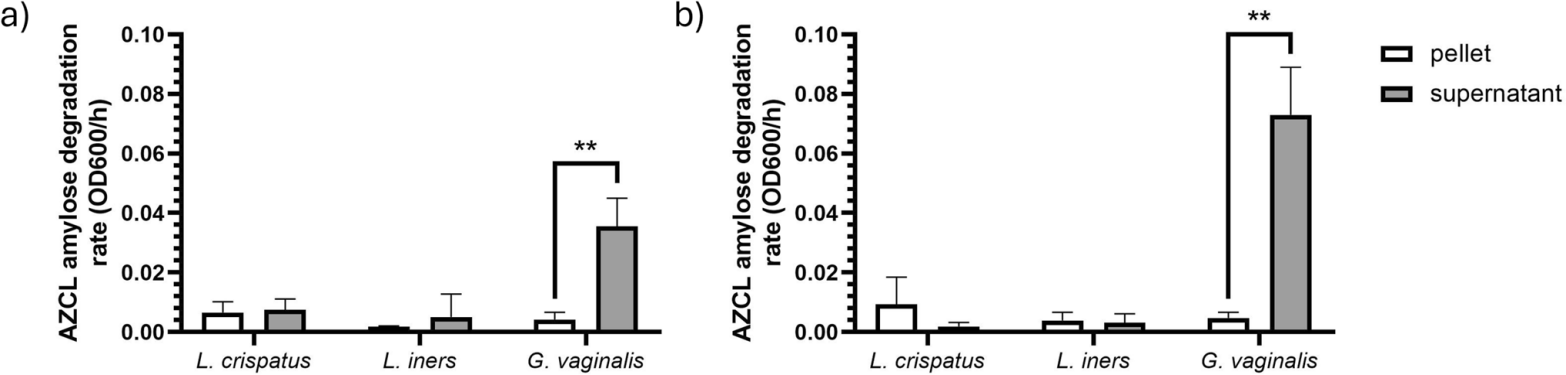
AZCL-amylose degradation rate expressed as the increase of optical density at 600 nm per hour in resuspended cell pellets (white bars) and supernatants (gray bars) corrected for optical density from *Lactobacillus crispatus* RL10 (*pulA*^+^), *Lactobacillus iners* ACS 049-v-Sch2 and *Gardnerella vaginalis* DSM 4944. Cultures were grown anaerobically in NYCIII medium with glycogen (panel **a**) or glucose (panel **b**) for 48 h. Bars represent the mean, and error bars represent standard deviation of three independent biological replicates. Pellets and supernatants were compared using t test. ** = *P*  < 0.005

**Fig. 3:**
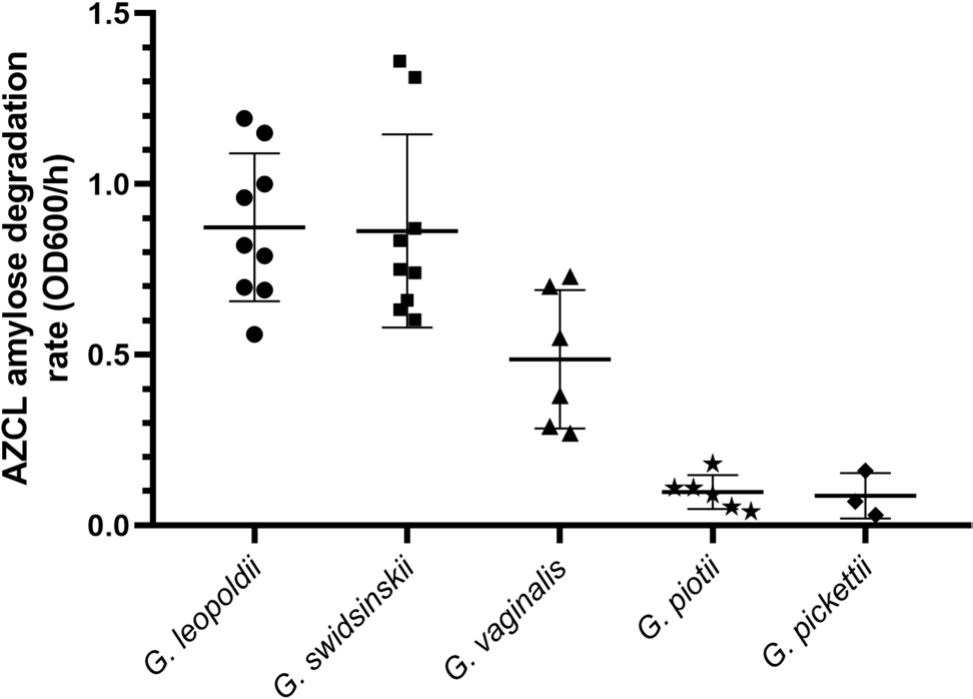
AZCL-amylose degradation rate by optical density increase at 600 nm of cultures (cells in growth medium) of *Gardnerella* species isolated by human vaginal samples grown for 48 h on NYC III medium with glucose. The degradation rate was corrected for cell density (OD600). Data points represent biological replicates

**Fig. 4:**
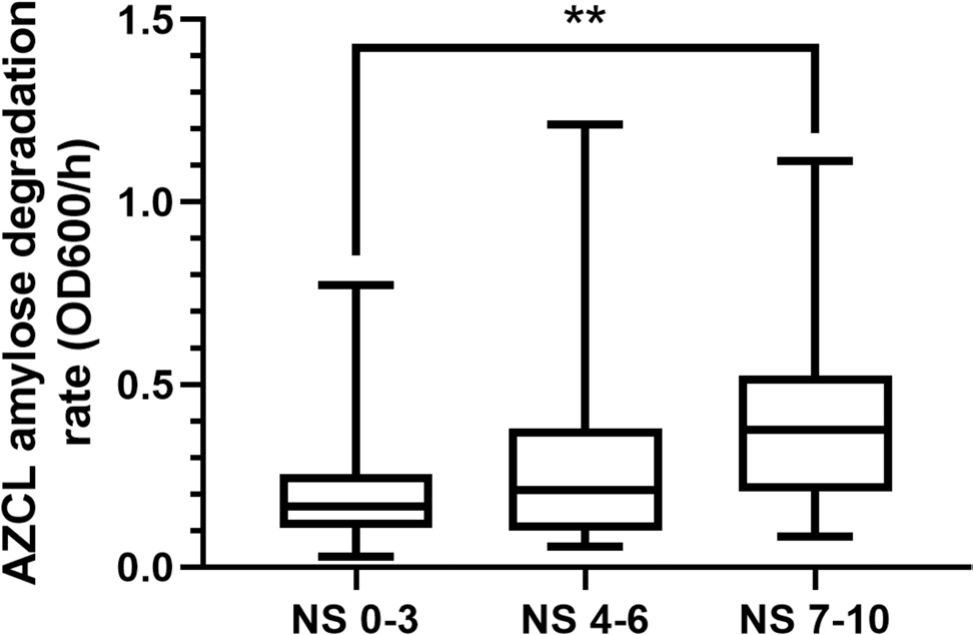
AZCL-amylose degradation rate of 96 vaginal samples with *Lactobacillus*-dominant microbiome (Nugent score 0–3), intermediate microbiome (Nugent score 4–6) or bacterial vaginosis (Nugent score 7–10). *P* values were calculated by ANOVA Tukey’s test.** = *P* < 0.005. Horizontal bars indicate the mean

For this study, we selected various strains isolated from vaginal samples. The isolation and characterization of the *Lactobacillus crispatus* amylopullulanase-sufficient strain RL10 and amylopullulanase-deficient strain RL09 were previously described [3, 4, 6]. Various *Gardnerella* strains were included as well as other members of the vaginal microbiota as described in Table S1. Cultivation of bacteria was carried out in laboratories of Ghent University and Vrije Universiteit Amsterdam – see below. The results from cultivation at the VU Amsterdam laboratory are depicted in Figs. 1, 2, 4, S1, and S2. The results from cultivation experiments at Ghent University are depicted in Fig. 3 and S3. All strains were anaerobically cultivated from − 80 °C glycerol stocks (VU Amsterdam: NYCIII medium + 20% glycerol, Ghent: TSB + 15% glycerol) on agar plates under conditions indicated in Table S1. From these plates, colonies were picked to inoculate liquid New York City (NYC)-III broth with 10% horse serum as described previously [3] or YEPG broth for *Candida albicans*. All cultures were handled under anaerobic conditions (VU Amsterdam: N_2_ + 10% CO_2_, Ghent University: 10% H_2_, 10% CO_2_, 80% N_2_) in an anaerobic chamber (VU Amsterdam: PLAS Labs 855 NB; Ghent university: Bugbox Plus).

For three common vaginal bacterial species—*Gardnerella vaginalis, Lactobacillus iners,* and *Lactobacillus crispatus*—we studied growth on glycogen by preparing a 1.1 × New York City (NYC)-III broth with 10% horse serum to which we added a stock solution of 50 g/L oyster glycogen (Alva Aesar, Haver Hill, MA, USA) or 55 g/L glucose-monohydrate (Sigma-Aldrich) to obtain a final concentration of 5 g/L glucose-equivalents. We compared optical density after anaerobic incubation in NYCIII medium on these media. To verify whether any other medium components resulted in growth, we inoculated these species also in NYCIII medium without glucose or glycogen supplemented. In Amsterdam, the optical density of cultures after 48 h of growth was determined by diluting in saline and measuring the turbidity at 600 nm with a 1-mL cuvette using a Varian Cary 50 Bio UV–Vis Spectrophotometer (Spectra Lab Scientific Inc, ON, Canada), subtracting the cell-free medium control. In Ghent, the optical density was measured by diluting the culture in phosphate-buffered saline in a 96-well microtiter plate and measuring the turbidity at 600 nm in a GloMax® Explorer Multimode microplate reader (Promega, Madison, WI, USA).

### The AZCL-Amylose Assay

High purity dyed and cross-linked insoluble AZCL-Amylose (product code O-AZAMYF; Megazyme Inc., Bray, Ireland) was used as substrate for the assay of α-amylase. The AZCL-Amylose was used in a raw ungelatinized form and suspended in an amylase buffer with xanthan gum to disperse the granules (Figure S1). After mixing with the sample (either vaginal sample of bacterial culture, spent medium or resuspended cell pellet), the increase in optical density in a microtiter plate was measured at 600 nm at 37 °C. The lack of heating or other pretreatment results in the substrate retaining a coarse, granular, undissolved structure. Pelleting was prevented in this assay by suspension in a viscous buffer containing xanthan gum. Xanthan gum (Sigma-Aldrich Life Corporation, Saint Louis, MI, USA) was dissolved at 5 mg/mL in amylase buffer (100 mM sodium acetate buffer containing 5 mM CaCl_2_, pH adjusted with HCl to 5.3). This solution was autoclaved at 121 °C for 20 min and kept at room temperature. AZCL-amylose is commercially available in two forms: regular and fine. The granules in the ‘fine’ product are small enough to pipet with regular pipet tips to dose and mix with the sample. This solution was stable without sedimentation of the raw amylose granules for up to 48 h. AZCL-amylose was dispersed in the xanthan gum/amylase buffer prior to each experiment by shortly vortexing. The assay was carried out in a 96-well plate covered with adhesive film. A total of 190 µL reagent solution was added to 10 µL sample and mixed by carefully pipetting up and down. The microplate was incubated at 37 °C for 24 h, and absorption was measured at 600 nm every 10 min. Maximum rate was taken as the maximum slope of a three-hour time frame (including 19 data points). This rate was divided by the optical density at 600 nm of the culture. The enzyme assay was validated using *Aspergillus oryzae* amylase (Sigma-Aldrich) solution in amylase buffer with 150 units/µL where one unit corresponds to the amount of enzyme needed to cleave 1 µmol of maltose per minute at pH 6.0 and 25 °C.

To study the prevalence of raw amylose degradation activity among *Gardnerella* species, we screened a set of 14 human vaginal *Gardnerella* strains from the species *G. leopoldii, G. swidsinskii, G. vaginalis, G. piotii,* and *G. pickettii.* We compared these to strains of other common species colonizing the human vagina including *Candida albicans*, *Fannyhessea vaginae* (previously known as *Atopobium vaginae*), *Prevotella bivia, Bifidobacterium,* and several *Lactobacillus* species. Cultures were either used without centrifugation or centrifuged for 1 min at 20,000 rcf at room temperature to separate cells. Spent medium was aspirated and mixed with the substrate, and cell pellets were resuspended in sterile phosphate-buffered saline and mixed with the substrate. The degradation rate was divided by the optical density at 600 nm.

### The AZCL-Amylose Assay and Quantification of Bacteria in Vaginal Swabs

Vaginal swabs were collected from patients visiting the Center for Sexual Health (Amsterdam, The Netherlands), as described previously [6]. The swabs were eluted in Amies buffer and 15% glycerol and cysteine were added for cryoprotection to preserve live bacteria and enzymes for subsequent isolation and analysis. The samples were stored at − 80 °C. Samples for this study were selected on the basis of Nugent scores choosing one third of samples (32 samples for each category) with scores between 0 and 3 (healthy), one third with scores between 4 and 6 (intermediate), and one third with a Nugent score between 7 and 10 (bacterial vaginosis), totaling 96 samples overall. For the amylose assay, samples were thawed, vortexed, and pipetted but not homogenized or centrifuged, which means that mucus and other solid material was still present in the sample when pipetted. Of the liquid fraction, 10 µL was transferred to a microtiter plate to which 190 µL of the AZCL-amylose in the amylase buffer with xanthan gum was added. The assay and rate calculation were carried out as described above.

The commercially available multiplex PCR for ATRiDA test BV diagnosis (ATRiDa, Amersfoort, The Netherlands) was performed according to instructions of the manufacturer ([32, 33] on isolated DNA from vaginal samples targeting *Lactobacillus* spp., total *Gardnerella*, total *Fannyhessea,* and total bacteria. Both the DNA extraction as well as the qPCR reaction were performed as described previously [34]. The same DNA extracts were utilized for species-specific *G. vaginalis*, *G. piotii*, *G. leopoldii,* and *G. swidsinskii* qPCR assays [35]. To account for multiple testing, we adjusted the *p* value threshold by Bonferroni-correction leading to a required *p* value of 0.013 (*P* = 0.05 divided by four outcomes) for the BV diagnostic PCR and the *Gardnerella* species-specific qPCR. We used the log transformed data to find correlations between AZCL-amylose degradation rate and abundance of any of the targets. All data processing and statistical analysis were performed in Microsoft Excel and GraphPad Prism 9.5.0.

## Results

### *Gardnerella *and *Lactobacillus iners* Can Use Glycogen as a Carbon Source for Growth

The optical density of *L. crispatus*, *L. iners,* and *G. vaginalis* after anaerobic growth in NYCIII medium with glucose or glycogen is shown in Fig. 1. As expected and previously shown [3, 4], the amylopullulanase-deficient strain of *L. crispatus* did not grow on glycogen, whereas the amylopullulanase-sufficient strain grew to a comparable optical density on glycogen as on glucose. The *L. iners* strain showed an equal optical density on glycogen compared to glucose, while the *G. vaginalis* strains showed a higher optical density on glycogen compared to glucose. To verify that the increase in turbidity was solely due to growth on the carbohydrate source added (glucose or glycogen), we added a control—the NYC medium without any glucose or glycogen. No increase in optical density was observed in this medium compared to the cell-free control (Fig. 1).

### Multi-well Assay with Undissolved Labeled Amylose to Measure Enzymatic Rate of α-Glucosidases

Next, we further characterized α-glucosidase activity of *L. crispatus*, *L. iners,* and G. *vaginalis* using a quantitative amylase assay with raw amylose labeled with a covalently linked azure cross-linked (AZCL) chromogenic substrate [36] in a microtiter plate. This chromogenic substrate is conventionally used to study amylase activity using agarose plates or in dissolved form but is applied here using undissolved granules in a xanthan gum buffer to keep the substrate dispersed. The release rate of blue dye is dependent on the enzymatic digestion rate of the labeled glycan (see calibration curve in Figure S2). No pretreatment, purification or dialysis of the sample is required, and the assay is suitable for use in multi-well format in a plate reader as well as for visual detection.

### *Gardnerella vaginalis* Shows Extracellular Amylose-Degrading Activity

In Fig. 2, the enzymatic activity in pellets and supernatants as determined by the amylose degradation assay is shown. The amylose degradation assay showed that the investigated *G. vaginalis* strain was capable of degrading the raw amylose substrate, while in *L. crispatus* and *L. iners* cultures, no labeled amylose digestion was observed. The activity was mostly found in supernatants of *G. vaginalis* compared to the cell pellet (*P* = 0.005)*.* The activity was also present in *G. vaginalis* culture grown on medium with glucose, indicating that *G. vaginalis* α-glucosidase activity is not subject to carbon-catabolite repression, as observed for the glycogen-degrading activity of *L. crispatus*. Similarly to what was observed in glycogen growth, also for glucose the supernatant was more active compared to the pellet (*P* = 0.002).

### *Gardnerella* Species Show Variation in AZCL-Amylose Degradation Rate

AZCL-amylose degradation activity of different *Gardnerella* species is shown in Fig. 3. Overall, the data obtained indicate a clear difference in the ability to degrade amylose among certain *Gardnerella* species. Using Tukey’s test for multiple comparisons, *G. leopoldii* showed highly significant differences compared to *G. piotii* (*P* < 0.0001) and *G. pickettii* 3 (*P* < 0.0001), with mean differences in amylose degradation rates of 0.776 and 0.787, respectively. Similarly, *G. swidsinskii* exhibited a significant difference compared to *G. piotii* (*P* < 0.0001) and *G. pickettii* (*P* < 0.0001). On the other hand, some comparisons were not statistically significant, such as between *G. leopoldii* and *G. swidsinskii* (*P* = 0.99) and between *G. vaginalis* and *G. pickettii* (*P* = 0.08).

### AZCL-Amylose Degradation Rate is Associated with Nugent Score

The substantial differences in amylose degradation activity between three of the most common vaginal species prompted us to study the presence of this enzymatic activity in vaginal swabs. The degradation rate in clinical samples across Nugent categorization is shown in Fig. 4. Degradation of amylose was strongly associated with Nugent scoring. Samples from women with Nugent score between 7 and 10 (bacterial vaginosis) showed a mean AZCL-amylose degradation rate of 0.41 OD_600_/h ± 0.27 compared to 0.22 OD_600_/h ± 0.18 in women with Nugent score 0–3 (*P* = 0.0051). The results of the qPCR for the genera *Gardnerella, Fannyhessea* and total bacteria were strongly associated with Nugent score. These species had a higher abundance in samples with Nugent score 7–10 compared to samples with a Nugent score of 0–3, while lactobacilli were more abundant in women with low Nugent scores compared to women with high Nugent scores. All correlations between the genera and Nugent score group are shown in Table S2.

In growth experiments, a higher AZCL-amylose degradation rate was observed in cultures of *Gardnerella* species compared to *Lactobacillus* species. Therefore, we hypothesized that the observed difference in samples from women with high Nugent scores compared to women with low Nugent scores may be due to higher *Gardnerella* abundance in the high Nugent score group. We therefore assessed whether the measured amylose degradation rate was associated with *Gardnerella* abundance and found a positive correlation between AZCL-amylose degradation rate and levels of *Gardnerella* and *Fannyhessea* (Spearman correlation of 0.24 and 0.23, *p* value of 0.016 and 0.028, respectively). The highest degradation rates were found in samples containing multiple *Gardnerella* species, including *G. leopoldii*, G*. swidsinskii,* and *G. piotii* (Figure S3).

### AZCL-Amylose Degradation and Sialidase Activity in *Gardnerella* Species

**Fig. 5:**
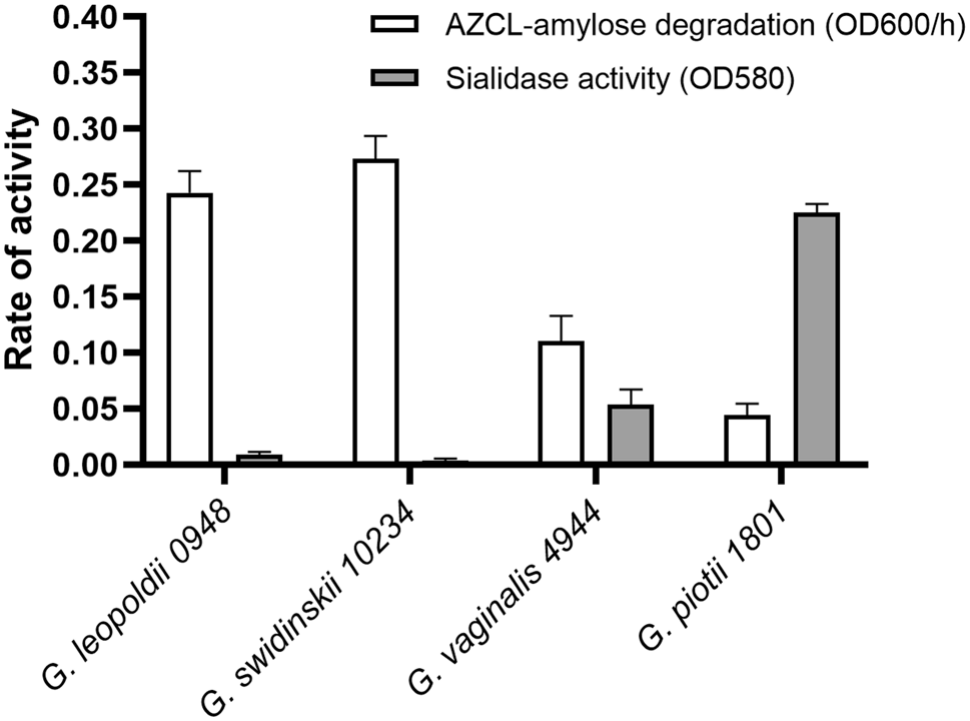
Amylose degradation rates and sialidase activity in supernatants of four *Gardnerella* species

Four strains of *Gardnerella*, each representing a distinct species, were selected for comparative analysis. After 48 h of growth, culture supernatants were harvested to assess AZCL-amylose degradation and sialidase activity. *G. swidsinskii* and *G. leopoldii* demonstrated higher AZCL-amylose degradation activity relative to *G. vaginalis* and *G. piotii*. Notably, pronounced sialidase activity was observed only in *G. piotii* strain 1801, consistent with the presence of the *nanH3* gene [37]. *G. vaginalis* exhibited intermediate sialidase activity (Fig. 5).

## Discussion

In this study, we investigated the presence of extracellular α-glucosidase activity among key members of the vaginal microbiota. These enzymes catalyze the breakdown of α1–4 and/or α1–6 glycosidic linkages in α-glucans such as glycogen, amylopectin, and amylose. Between puberty and menopause, the female reproductive tract contains substantial glycogen reserves [5], which may serve as a carbon source for bacteria that express and secrete glycogen-degrading enzymes. Previously, we demonstrated that *Lactobacillus crispatus* strains can degrade starch and glycogen [3, 4]. Jenkins et al. identified and characterized the amylopullulanase responsible for this activity in *L. crispatus* [30]. However, in the current study, we found that *L. crispatus* was unable to degrade raw amylose—composed primarily of α1–4 linkages—whereas *Gardnerella* species demonstrated a clear capacity to do so. Previously, resistant starches were found to modulate the gut microbiome [26–29]. High amylose corn starch is degraded by specific gut genera, one of which is *Bifidobacterium* that is closely related to the genus *Gardnerella*. The amylopullulanase of bifidobacteria has the same carbohydrate-binding, amylase and pullulanase domains, N-terminal signal peptide, and C-terminal transmembrane domain as the *Gardnerella* enzyme that is likely responsible for the observed raw amylose degradation activity in this study [23, 30]. Enzymes with such a domain structure are typically classified in the α-amylase superfamily (GH13 and GH57) and often contain starch-binding modules. The non-catalytic carbohydrate-binding modules (CBMs) target the enzyme to insoluble starch or glycogen, and the presence of multiple CBM domains can further enhance binding to resistant starch, facilitating more efficient degradation [21, 23]. A major limitation in our study is that we inferred the enzyme’s identity based on activity and homology, but did not directly confirm the specific protein in our experiments. However, preliminary research identified five peptides matching the *Gardnerella* pullulanase enzyme (protein accession S4H1S5; 2014 AA) in digested protein fractions exhibiting amylose-degrading activity, but we did not succeed in purifying the enzyme to homogeneity.

Interestingly, we observed clear differences between *L. crispatus* and *G. vaginalis* α-glucosidase activity not only in substrate specificity but also in its regulation and localization. In contrast to *L. crispatus*, which exhibits catabolite repression of amylopullulanase in the presence of glucose and maltodextrins [3], *Gardnerella vaginalis* retained α-glucosidase activity even when grown on glucose. Additionally, while *L. crispatus* amylase activity is largely cell-associated, the *Gardnerella* activity was primarily extracellular, despite the presence of a C-terminal transmembrane domain. It is possible that the physical structure of our chemically labeled amylose substrate limited access for cell-bound enzymes, or that proteolytic cleavage of the transmembrane domain releases the enzyme into the environment. This phenomenon mirrors what has been described for *Gardnerella* sialidase, which is also partially released into culture supernatants despite having a membrane anchor [16]. Future experiments are required to precisely localize these enzymes and investigate post-translational cleavage.

Although AZCL-amylose degradation activity correlated well with Nugent scores, we did not observe a clear association with the abundance of any single *Gardnerella* species. For example, *G. swidsinskii*-dominant samples exhibited both high and low degradation activity, and several high-activity samples lacked detectable *G. swidsinskii* or *G. leopoldii*. However, the highest degradation rates were found in samples containing multiple *Gardnerella* species (*G. leopoldii*, *G. swidsinskii*, and *G. piotii*). These findings suggest strain-level variation within species, and potential cooperative interactions when multiple species are present. While host-derived amylase has been detected in vaginal fluid and proposed to contribute to glycogen degradation [24, 25, 38], our data suggest that its role may be limited in *Lactobacillus*-dominated communities, given the generally low amylose degradation activity in samples with Nugent scores of 0–3. We cannot exclude a contribution of human amylase in BV-associated communities, but the absence of detectable degradation by other BV-associated genera (e.g., *Fannyhessea*, *Prevotella*, and vaginal *Bifidobacterium*) leaves *Gardnerella* as the most likely microbial source. However, this limitation in our study could be addressed in future studies by using specific inhibitors to quantify the contribution of host-derived enzymes.

To contextualize *Gardnerella*’s α-glucosidase activity within a broader enzymatic profile, we also measured sialidase activity. Our data suggest division of labor among species: *G. swidsinskii* and *G. leopoldii* exhibited higher amylose-degrading activity compared to *G. vaginalis* and *G. piotii*, while *G. piotii* was the only strain with notable sialidase production. This is consistent with prior reports linking bacterial vaginosis (BV) to increased species diversity within the *Gardnerella* genus rather than specific *Gardnerella* species [39]. While our data suggest a potential trend between amylase and sialidase activity across species, the limited sample size precludes any definitive conclusions regarding an inverse correlation.

Taken together, the characteristics of the *Gardnerella* α-glucosidase could explain the low glycogen levels in samples with BV [5]. Lactobacilli may in contrast only ‘graze’ the accessible linkages of the glycogen molecules and cease amylopullulanase expression as soon as the smaller maltodextrins start to accumulate [3]. Previously, *Gardnerella* sialidase and cytolysin were found to be linked to clinical characteristics of BV, namely mucus thinning and epithelial exfoliation [14, 15]. Here, we propose that *Gardnerella*’s glycogen metabolism represents a third clinical characteristic of BV. This activity links its colonization and overgrowth to the reduced glycogen levels observed in the vaginal lumen during BV. Understanding the enzymatic capabilities and interspecies variation of *Gardnerella* may be crucial to further unravel its role in BV pathophysiology.

The ability of *Gardnerella* species to secrete extracellular enzymes that degrade resistant glycogen structures such as amylose highlights a potential microbial mechanism contributing to glycogen depletion in BV. These findings suggest that targeting bacterial α-glucosidase activity—specifically the amylase-like enzymes of *Gardnerella*—could represent a novel therapeutic strategy for the prevention or treatment of BV. Inhibiting this activity may help preserve vaginal glycogen levels, thereby supporting the growth of beneficial lactobacilli and promoting a more stable, protective microbial community. Future work should explore the feasibility and specificity of such targeted interventions.

## Supplementary Information

Below is the link to the electronic supplementary material.

Supplementary file1 (XLSX 602 KB)

Supplementary file2 (DOCX 333 KB)

## References

[1] Cruickshank R (1934) The conversion of the glycogen of the vagina into lactic acid. J Pathol Bacteriol. 10.1002/path.1700390118

[2] Mirmonsef P, Hotton AL, Gilbert D et al (2016) Glycogen levels in undiluted genital fluid and their relationship to vaginal pH, estrogen, and progesterone. PLoS ONE 11:e0153553. 10.1371/journal.pone.0153553 27093050 10.1371/journal.pone.0153553 PMC4836725

[3] Hertzberger R, May A, Kramer G et al (2022) Genetic elements orchestrating *Lactobacillus crispatus* glycogen metabolism in the vagina. Int J Mol Sci 23:5590. 10.3390/ijms23105590 35628398 10.3390/ijms23105590 PMC9141943

[4] van der Veer C, Hertzberger RY, Bruisten SM et al (2019) Comparative genomics of human *Lactobacillus crispatus* isolates reveals genes for glycosylation and glycogen degradation: implications for in vivo dominance of the vaginal microbiota. Microbiome 7:49. 10.1186/s40168-019-0667-9 30925932 10.1186/s40168-019-0667-9 PMC6441167

[5] Mirmonsef P, Hotton AL, Gilbert D et al (2014) Free glycogen in vaginal fluids is associated with *Lactobacillus* colonization and low vaginal pH. PLoS ONE 9:e102467. 10.1371/journal.pone.0102467 25033265 10.1371/journal.pone.0102467 PMC4102502

[6] Dols JAM, Molenaar D, van der Helm JJ et al (2016) Molecular assessment of bacterial vaginosis by *Lactobacillus* abundance and species diversity. BMC Infect Dis. 10.1186/s12879-016-1513-3 27107961 10.1186/s12879-016-1513-3 PMC4841971

[7] Gosmann C, Anahtar MN, Handley SA et al (2017) *Lactobacillus*-deficient cervicovaginal bacterial communities are associated with increased HIV acquisition in young South African women. Immunity 46:29–37. 10.1016/j.immuni.2016.12.013 28087240 10.1016/j.immuni.2016.12.013 PMC5270628

[8] Lin D, Kouzy R, Abi Jaoude J et al (2020) Microbiome factors in HPV-driven carcinogenesis and cancers. PLoS Pathog 16:e1008524. 10.1371/journal.ppat.1008524 32497113 10.1371/journal.ppat.1008524 PMC7271998

[9] Vergaro P, Tiscornia G, Barragán M et al (2019) Vaginal microbiota profile at the time of embryo transfer does not affect live birth rate in IVF cycles with donated oocytes. Reprod Biomed Online 38:883–891. 10.1016/j.rbmo.2018.12.019 30879910 10.1016/j.rbmo.2018.12.019

[10] Haggerty CL, Hillier SL, Bass DC et al (2004) Bacterial vaginosis and anaerobic bacteria are associated with endometritis. Clin Infect Dis 39:990–995. 10.1086/423963 15472851 10.1086/423963

[11] Brown RG, Al-Memar M, Marchesi JR et al (2018) Establishment of vaginal microbiota composition in early pregnancy and its association with subsequent preterm prelabour rupture of the fetal membranes. Transl Res. 10.1016/j.trsl.2018.12.005 30633889 10.1016/j.trsl.2018.12.005 PMC6489901

[12] Gudnadottir U, Debelius JW, Du J et al (2022) The vaginal microbiome and the risk of preterm birth: a systematic review and network meta-analysis. Sci Rep 12:7926. 10.1038/s41598-022-12007-9 35562576 10.1038/s41598-022-12007-9 PMC9106729

[13] Gelber SE, Aguilar JL, Lewis KLT, Ratner AJ (2008) Functional and phylogenetic characterization of vaginolysin, the human-specific cytolysin from *Gardnerella vaginalis*. J Bacteriol 190:3896–3903. 10.1128/JB.01965-07 18390664 10.1128/JB.01965-07 PMC2395025

[14] Gilbert NM, Lewis WG, Lewis AL (2013) Clinical features of bacterial vaginosis in a murine model of vaginal infection with *Gardnerella vaginalis*. PLoS ONE 8:e59539. 10.1371/journal.pone.0059539 23527214 10.1371/journal.pone.0059539 PMC3602284

[15] Lewis WG, Robinson LS, Gilbert NM et al (2013) Degradation, foraging, and depletion of mucus sialoglycans by the vagina-adapted *Actinobacterium Gardnerella vaginalis*. J Biol Chem 288:12067–12079. 10.1074/jbc.M113.453654 23479734 10.1074/jbc.M113.453654 PMC3636892

[16] Robinson LS, Schwebke J, Lewis WG, Lewis AL (2019) Identification and characterization of NanH2 and NanH3, enzymes responsible for sialidase activity in the vaginal bacterium *Gardnerella vaginalis*. J Biol Chem 294:5230–5245. 10.1074/jbc.RA118.006221 30723162 10.1074/jbc.RA118.006221 PMC6462536

[17] Sousa M, Ksiezarek M, Perovic SU et al (2023) Gardnerella pickettii sp. nov. (formerly Gardnerella genomic species 3) and Gardnerella greenwoodii sp. nov. (formerly Gardnerella genomic species 8) isolated from female urinary microbiome. Int J Syst Evolut Microbiol. 10.1099/ijsem.0.006140 10.1099/ijsem.0.006140 37921436

[18] van der Maarel MJEC, van der Veen B, Uitdehaag JCM et al (2002) Properties and applications of starch-converting enzymes of the alpha-amylase family. J Biotechnol 94:137–155. 10.1016/s0168-1656(01)00407-2 11796168 10.1016/s0168-1656(01)00407-2

[19] Janeček Š (2023) Advances in amylases—what’s going on? Molecules 28:7268. 10.3390/molecules28217268 37959687 10.3390/molecules28217268 PMC10647339

[20] Janeček Š, Gabriško M (2016) Remarkable evolutionary relatedness among the enzymes and proteins from the α-amylase family. Cell Mol Life Sci 73:2707–2725. 10.1007/s00018-016-2246-6 27154042 10.1007/s00018-016-2246-6 PMC11108405

[21] You Y, Kong H, Li C et al (2024) Carbohydrate binding modules: compact yet potent accessories in the specific substrate binding and performance evolution of carbohydrate-active enzymes. Biotechnol Adv 73:108365. 10.1016/j.biotechadv.2024.108365 38677391 10.1016/j.biotechadv.2024.108365

[22] Sycuro L, Lithgow K, Cochinamogulos A et al (2023) Glycogen-Degrading pullulanase a is variably present and correlated with lactic acid in vaginal samples from young African women. Am J Obstet Gynecol 228:S788. 10.1016/j.ajog.2022.11.148

[23] Bhandari P, Tingley J, Abbott DW, Hill JE (2023) Glycogen-degrading activities of catalytic domains of α-amylase and α-amylase-pullulanase enzymes conserved in Gardnerella spp. from the vaginal microbiome. J Bacteriol 205:e0039322. 10.1128/jb.00393-22 36744900 10.1128/jb.00393-22 PMC9945562

[24] Nunn KL, Clair GC, Adkins JN et al (2020) Amylases in the human vagina. mSphere 5:e00943-20. 10.1128/mSphere.00943-20 33298571 10.1128/mSphere.00943-20 PMC7729256

[25] Spear GT, French AL, Gilbert D et al (2014) Human alpha-amylase present in lower-genital-tract mucosal fluid processes glycogen to support vaginal colonization by *Lactobacillus*. J Infect Dis 210:1019–1028. 10.1093/infdis/jiu231 24737800 10.1093/infdis/jiu231 PMC4168305

[26] Brown I, Warhurst M, Arcot J et al (1997) Fecal numbers of *Bifidobacteria* are higher in pigs fed *Bifidobacterium longum* with a high amylose cornstarch than with a low amylose cornstarch. J Nutr 127:1822–1827. 10.1093/jn/127.9.1822 9278566 10.1093/jn/127.9.1822

[27] Sybille T, June Z, Michael K et al (2013) The intestinal microbiota in aged mice is modulated by dietary resistant starch and correlated with improvements in host responses. FEMS Microbiol Ecol 83:299–309. 10.1111/j.1574-6941.2012.01475.x 22909308 10.1111/j.1574-6941.2012.01475.x

[28] Jung D-H, Seo D-H, Kim Y-J et al (2020) The presence of resistant starch-degrading amylases in *Bifidobacterium adolescentis* of the human gut. Int J Biol Macromol 161:389–397. 10.1016/j.ijbiomac.2020.05.235 32479932 10.1016/j.ijbiomac.2020.05.235

[29] Maier TV, Lucio M, Lee LH et al (2017) Impact of dietary resistant starch on the human gut microbiome, metaproteome, and metabolome. MBio 8:e01343–17. 10.1128/mBio.01343-17 29042495 10.1128/mBio.01343-17 PMC5646248

[30] Jenkins DJ, Woolston BM, Hood-Pishchany MI et al (2023) Bacterial amylases enable glycogen degradation by the vaginal microbiome. Nat Microbiol 8:1641–1652. 10.1038/s41564-023-01447-2 37563289 10.1038/s41564-023-01447-2 PMC10465358

[31] Bhandari P, Hill JE (2023) Transport and utilization of glycogen breakdown products by *Gardnerella* spp. from the human vaginal microbiome. Microbiol Spectr 11:e04435-22. 10.1128/spectrum.04435-22 36920187 10.1128/spectrum.04435-22 PMC10101108

[32] Rumyantseva T, Shipitsyna E, Guschin A, Unemo M (2016) Evaluation and subsequent optimizations of the quantitative AmpliSens florocenosis/bacterial vaginosis-FRT multiplex real-time PCR assay for diagnosis of bacterial vaginosis. APMIS 124:1099–1108. 10.1111/apm.12608 27714844 10.1111/apm.12608

[33] Shipitsyna E, Khusnutdinova T, Budilovskaya O et al (2020) Bacterial vaginosis-associated vaginal microbiota is an age-independent risk factor for *Chlamydia trachomatis*, *Mycoplasma genitalium* and *Trichomonas vaginalis* infections in low-risk women, St. Petersburg, Russia. Eur J Clin Microbiol Infect Dis 39:1221–1230. 10.1007/s10096-020-03831-w 32036466 10.1007/s10096-020-03831-w PMC7303053

[34] van der Veer C, Houdt R, Van Dam A, Bruisten S (2018) Accuracy of a commercial multiplex PCR for the diagnosis of bacterial vaginosis. J Med Microbiol. 10.1099/jmm.0.000792 29985123 10.1099/jmm.0.000792 PMC6230723

[35] Latka A, Van Simaey L, Reynders M et al (2022) Optimization of Propidium monoazide qPCR (viability-qPCR) to quantify the killing by the *Gardnerella*-specific endolysin PM-477, directly in vaginal samples from women with bacterial vaginosis. Antibiotics 11:111. 10.3390/antibiotics11010111 35052988 10.3390/antibiotics11010111 PMC8773202

[36] McCleary BV (1980) New chromogenic substrates for the assay of alpha-amylase and (1→4)-β-d-glucanase. Carbohydr Res 86:97–104. 10.1016/S0008-6215(00)84584-X 6159974 10.1016/s0008-6215(00)84584-x

[37] Kurukulasuriya SP, Patterson MH, Hill JE (2021) Slipped-strand mispairing in the gene encoding sialidase NanH3 in *Gardnerella* spp. Infect Immun 89:e00583-20. 10.1128/IAI.00583-20 33361200 10.1128/IAI.00583-20 PMC8097274

[38] Nasioudis D, Beghini J, Bongiovanni AM, et al (2015) Alpha-amylase in vaginal fluid: association with conditions favorable to dominance of Lactobacillus. Reproductive sciences (Thousand Oaks, Calif) 10.1177/1933719115581000 25878210

[39] Munch MM, Strenk SM, Srinivasan S et al (2024) *Gardnerella* species and their association with bacterial vaginosis. J Infect Dis 230:e171–e181. 10.1093/infdis/jiae026 39052736 10.1093/infdis/jiae026 PMC11272073

